# Synthesis of Arbitrary Quantum Circuits to Topological Assembly

**DOI:** 10.1038/srep30600

**Published:** 2016-08-02

**Authors:** Alexandru Paler, Simon J. Devitt, Austin G. Fowler

**Affiliations:** 1 Universitatea Transilvania, Facultatea de Matematică si Informatică, Braşov 500091, România; 2 Center for Emergent Matter Sciences, Riken, Saitama 351-0198, Japan; 3 Google Inc., Santa Barbara, California 93117, USA

## Abstract

Given a quantum algorithm, it is highly nontrivial to devise an efficient sequence of physical gates implementing the algorithm on real hardware and incorporating topological quantum error correction. In this paper, we present a first step towards this goal, focusing on generating correct and simple arrangements of topological structures that correspond to a given quantum circuit and largely neglecting their efficiency. We detail the many challenges that will need to be tackled in the pursuit of efficiency. The software source code can be consulted at https://github.com/alexandrupaler/tqec.

## Motivation

Quantum computers are envisioned to become reality, and a vast amount of research has been devoted to the theoretical foundations of this emerging computing paradigm as well as to investigating and developing initial prototypical hardware to support it. Any practical quantum computer should be able to reliably solve interesting computational problems, but reliability is one of the key engineering challenges.

It has been shown theoretically that it is possible to execute scalable (arbitrarily long) quantum computations on hardware with a failure probability below a certain threshold by using a sufficiently capable error-correcting code. A class of the most promising error-correcting codes, based on *topological cluster states*, enables scalable computations for failure probabilities below 1%, which is considered an achievable threshold with the current technological state of the art. The advantage of knowing how to reliably implement quantum computations is counterbalanced by the high resources introduced by the error-correction. The challenge to reliably implement quantum computations is partially a problem of optimising quantum error-corrected quantum circuits. However, these have to be firstly obtained (synthesised) from non-error-corrected (non-fault-tolerant) versions.

This work introduces the *synthesis* of *topologically quantum error-corrected (TQEC) circuits*. TQEC circuits are error-corrected quantum circuits operating on information encoded into *topological cluster states*. Synthesis of such circuits requires decomposing (translating) an abstract, high-level circuit description of a computation into a form compatible with TQEC that can be implemented and optimised on real world hardware.

TQEC circuits have a intrinsic visual representation, and their functionality can be described entirely using geometric elements. TQEC synthesis outputs an *assembly* of geometrically abstracted elements which are understood as being protected against errors (faults) by codes using topological considerations. Without affecting the generality of the proposed synthesis method, this work will not present the error-correction mechanisms^1^, and instead focus on establishing a terminology to easily connect the existing theoretical work to future engineering problems.

In the following, the first section presents the essential prerequisites for introducing the synthesis method, and the methods and algorithms of TQEC synthesis are exemplified and discussed afterwards. Finally, future work is analysed and conclusions are formulated. All the terms necessary for a better contextualisation of this work are explained in the Appendix accompanying this work. The Appendix also offers technical details about how synthesised FTQC circuits are used during their execution.

## A Brief Introduction to TQEC

An introduction is required for presenting the proposed synthesis, and this section offers the necessary details. The introduction starts by showing that TQEC circuits operate at a *logical layer*, which is an abstraction of a physical one. The logical abstraction makes use of topological cluster states, which have the structure of a regular *lattice* of *physical qubits* entangled into a large *graph state*. Therefore, topological cluster states are used to introduce the elements that define the translation between the quantum circuit formalism and the TQEC circuit description. The specification of TQEC circuits is based on the *geometry* of the entities formed in the logical layer.

### Defects, Qubits and Gates

**Figure 1:**
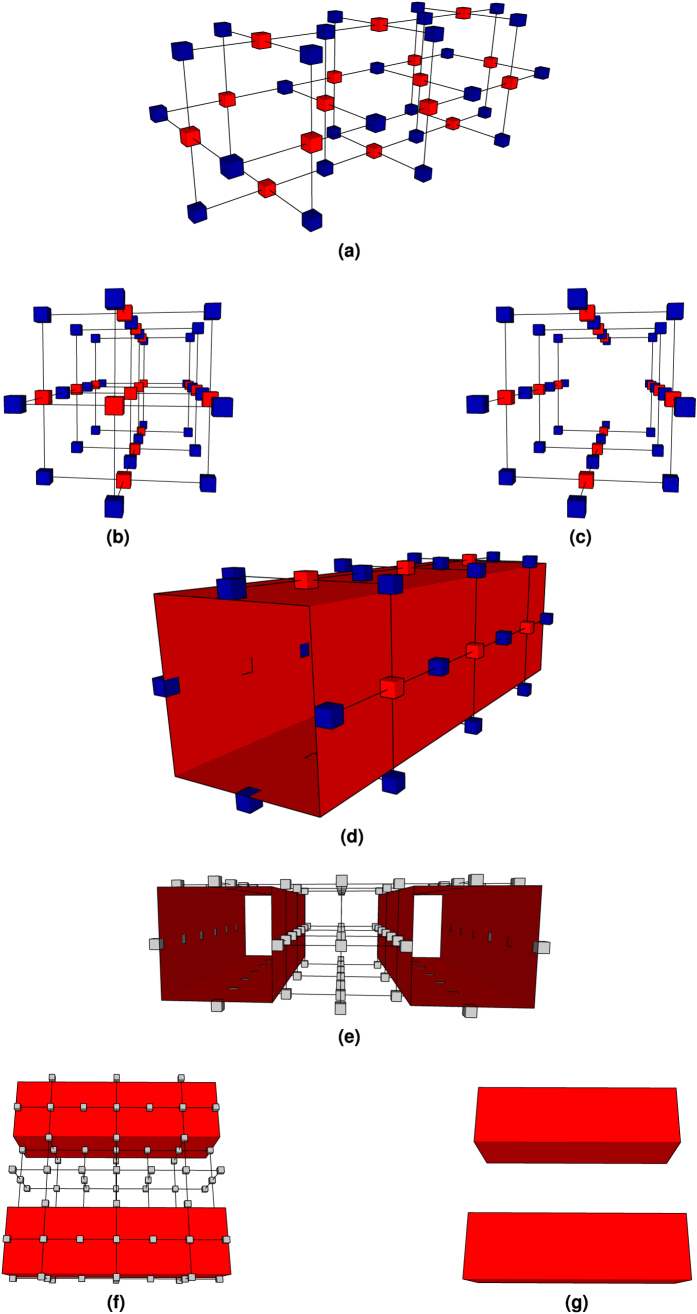
Construction of defects: (**a**) A lattice of three primal unit cells that will be used to construct a primal defect. For visualisation purposes, no dual unit cell is illustrated in the lattice. The red physical qubits represent primal qubits, and blue ones dual qubits. Removing a region of primal qubits creates a primal defect. Defects have a minimal diameter of one unit cell. (**b**) Rotated view of the previous figure. (**c**) Example of a defect constructed by removing four primal qubits from the graph. (**d**) The boundary of the defect can be abstracted by a cuboid (red). (**e**) Two defect example constructed in a graph consisting of 3 × 3 × 1 unit cells. Physical qubit types are not colour coded. (**f**) Rotated view of the previous figure. (**g**) The cuboids abstracting the defects can be themselves abstracted by segments whose end points are specified in terms of unit cell coordinates. The graph state used to encode logical information is thus not necessary for the specification of a TQEC circuit.

**Figure 2:**
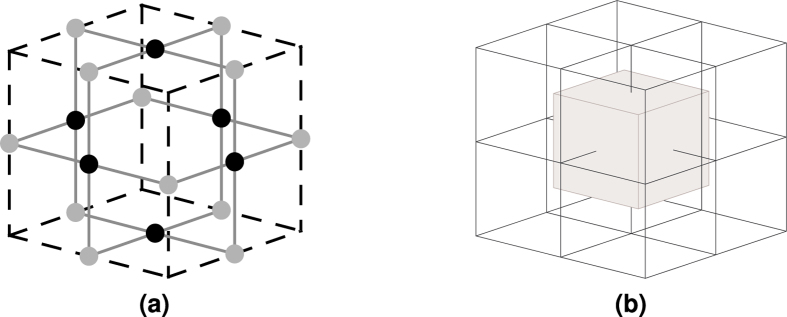
Unit cells: (**a**) The graph state required for TQEC is constructed using unit cells consisting of 18 physical qubits entangled according to the pattern presented in the figure. The face qubits are marked black, and the edge qubits are gray. Considering that the there are 27 possible positions in the three dimensional space used for illustrating the unit cell, and that the lowest coordinate is (0, 0, 0), then the cell centre has coordinate (1, 1, 1). Black qubits have an even coordinate, and grey qubits two even coordinates. (**b**) After stacking eight primal unit cells as in the figure, a unit cell will result in the middle of the 2 × 2 × 2 block. Such cells are called dual, and their centres have even coordinates.

Quantum information encoding into topological cluster states requires firstly the construction of a highly regular graph state, and secondly the removal of specific vertices from a lattice abstracting the state (Fig. 1)^2^. Each vertex represents a physical qubit, and edges stand for the entanglement relations between qubits. The removal of vertices results in the definition of *defects*. The lattice consists of two self-similar sublattices (*primal* and *dual*), where both are the result of stacking unit cells (Fig. 2a) along three axes. Their duality is perceived after stacking eight primal unit cells: a dual unit cell results at the geometric centre of a 2 × 2 × 2 (primal) unit cell block (Fig. 2b). The definition of a primal or dual defect depends on which sublattice vertices are removed from. A defect cannot have different types along its path, meaning that if one starts to define a primal defect, primal face qubits from the primal lattice will be removed until the defect ends. Due to practical error-correction reasons, a *logical* qubit is defined by a pair of same-type defects and, as a consequence, there are primal and dual (logical) qubits. Each defect consists of multiple linear *segments*.

**Figure 3:**
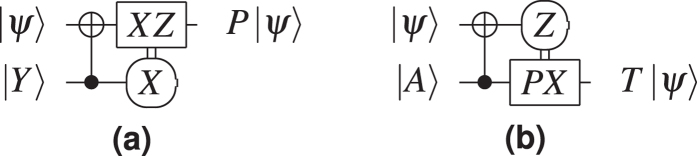
Teleported rotation gates using the states |*A*〉 and |*Y*〉. The correction for the *P*-gate is the Pauli *Y*-gate and can be tracked^9^, while the correction for the *T*-gate requires a subsequent *P*-gate that must be applied immediately.

Logical qubit types are relevant because the logical CNOT gate is implemented by *braiding* a dual defect around a primal one: the dual qubit always acts as control and the primal qubit as target. However, a CNOT is not sufficient for *computational universality* and more types of gates are required. For practical purposes, the gates supported by a quantum computing architecture form a discrete universal gate set, and TQEC circuits use {*CNOT, V, P, T*}. Single qubit *rotation gates* are implemented through teleportation-based mechanisms. Before teleportation, specific ancilla logical states (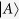 or 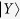) must be prepared. The implementation of the teleported T gate requires an 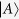 state initialised ancilla, and the teleported P and V gates use an ancilla initialised in 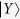 (Fig. 3). The first step when preparing logical ancillae is *state injection*: 1) initialising graph vertices (physical qubits) in those states, and then 2) constructing defects starting from the specially initialised vertices.


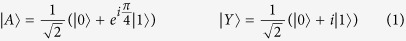


### Boxes

Injected states may have low *fidelity*^1^. State fidelity can be increased by replacing direct injections with the output of a *distillation circuit*. Such circuits take multiple low fidelity instances of injected states and output a single higher fidelity state.

Distillation procedures are probabilistic, meaning that it could happen that their output does not have the required fidelity. Thus, the *failure probability* of a TQEC rotation gate (T, P or V) is directly related to the failure probability of the box used to output the required ancilla state. In order to lower the failure probability, a larger number of boxes needs to accompany the circuit: if one of the distillations fails, a sufficient number of *spare* boxes is necessary for achieving a targeted lower TQEC gate failure probability.

Distillation circuits are frequently used due to the *ICM* form (single qubit initialisations, CNOT gates and single qubit measurements) of TQEC circuits (see Section “ICM Conversion”). Therefore, it is sufficient to use *boxes* as place holders for distillations in the TQEC circuit’s structure. There are two types of boxes, one for each 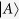 and 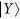 distillations.

### Pins and Connections

**Figure 4:**
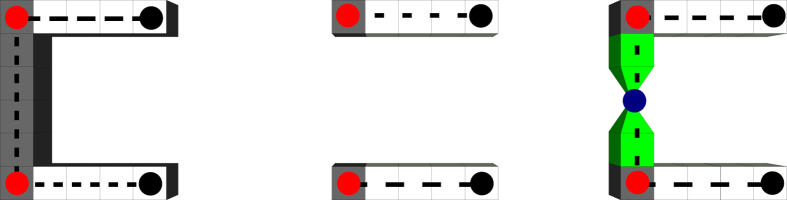
The geometries for initialising and measuring logical qubits. The grey segments represent the geometry to construct and join with the defects representing logical qubit segments (white). Each segment in this figure is defined using four unit cells. Dotted lines abstract defect segments, red dots are marking the pins, the black dots represent segment end points. The blue dot marks the graph vertex used for state injection. For primal logical qubits: left) Z-basis initialisation; centre) X-basis initialisation; right) state injection, where from a graph vertex two pyramid-shaped defects are constructed. For dual logical qubits: left) X-basis initialisation; centre) Z-basis initialisation. The measurement of a logical qubit uses the same initialisation geometries. Assuming that in the figures a time axis is represented horizontally from the left to the right, the measurement geometries will be mirrored against the vertical axis.

An arbitrary TQEC circuit has inputs and outputs (state injections are a particular case). Some of these are *configurable* (their defect structure is configurable), while others are not (e.g. teleportation ancillae or state injections). Such inputs/outputs are used to parameterise the computation implemented as a circuit, e.g. input numbers to an adder circuit. The difference between the input/output structures (Fig. 4) is a single defect segment which can be split into two non-disjoint segments sharing a common lattice vertex. Without loss of generality, the shared vertex is the geometric centre of the difference segment (cf. left and centre figures in Fig. 4). While for injected states the defects end right before the injection vertex, for configurable inputs/outputs the defects include the vertex (cf. right and left figures in Fig. 4). The only segments, where one of the end points refers to a vertex, are those in the direct neighbourhood of a state injection or configurable input/output. The other end point of such segments is abstracted using a *pin*. As a result, each injection and configurable input/output determine two pins (*pin pair*).

In general, pins are used for connecting circuits: the output pins of a circuit are *connected* to the input pins of another circuit. For example, the geometry of an adder can be chained multiple times in order to construct a multiplier: the outputs of the first adder will be connected to the inputs of the second one. Another example is when pins are used for connecting distillation boxes to the circuit: box output pins of distillation are connected to the injection pins of the circuit. In particular, TQEC circuit connections are implemented by defects.

### Geometric Description

TQEC synthesis generates the *geometric description* of a TQEC circuit^3^. The encoding (constructing defects) and the manipulation of information (braiding defects) is described independently from the initial lattice by concentrating entirely on the geometry of the defect structures. Geometries are a sufficient description also when considering that logical qubit initialisation and measurement basis are a function of defect configuration and logical qubit type (Fig. 4). Therefore, the positions of defect segments, inputs/outputs, injections and boxes are the elements of a geometric description.

A TQEC geometric description uses three dimensional coordinates because the lattice can be represented in three dimensions. Assuming all the coordinates are positive integers, and observing the lattice structure, it can be concluded that primal unit cell centres have odd coordinates, and dual unit cell centres have even coordinates (Fig. 2a). Vertices on the faces of a primal unit cell (primal face qubits) have an odd and two even coordinates. Consequently, vertices on the faces of a dual unit cell (dual face qubits) have a single even and two odd coordinates.

Defect segments have their end point coordinates specified using unit cell centre coordinates. State injections and configurable inputs/outputs are specified using vertex coordinates. The coordinates of the lattice vertex where a state is injected needs to specified too, because from that point starts the encoding of the logical qubit (the associated physical qubit is measured in the *X* basis). Pins (e.g. marked by red dots in Fig. 4) are specified using unit cell centre coordinates. Boxes abstract the bounding box of a distillation circuit geometric description. For each of the 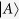 and 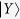 distillation procedures a particular box type with parametrised dimensions is used. The position of each box in the synthesised TQEC circuit is specified using unit cell centre coordinates.

The geometric description is independent of the code distance (consider distance *d*) and captures the TQEC computation. Therefore, instead of counting the number of lattice unit cells required to execute a geometrically described TQEC computation, a volume measure unit is used. The cubic *volume unit* is a standardised lattice with fixed dimensions (more details in the Appendix). The unit is obtained by tiling cubes instead of unit cells, where a cube with sides of length *d* contains *d*^3^ primal unit cells. Therefore, the complete lattice required for executing a TQEC circuit is the result of tiling volume units, which contain an exact number of cubes, which in turn are three dimensional tilings of unit cells. Volume units and cubes are intermediate abstractions between a complete lattice and unit cells. If one replaces every occurrence of unit cell with the term cube, the geometric description is valid for arbitrary error-correction procedures using topological cluster states.

## Synthesis of TQEC Circuits

The present method allows synthesising arbitrary quantum circuits, supports the placement and the diagrammatic representation of distillation subcircuits, and includes an automated method for connecting two distinct pins from the geometry. The TQEC synthesis presented in this work is more versatile than that of^4^, because it did not consider configurable inputs/outputs, and included a topological implementation of the logical CNOT which is different from braiding. The current synthesis was implemented and its source code can be consulted^5^. The method consists of multiple steps described in the following.

### Gate Decomposition

**Figure 5:**
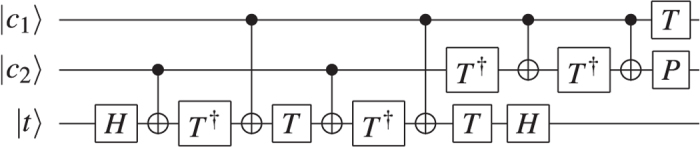
Toffoli gate implemented by a sequence of CNOT, *T, T*^†^, *P* and *H * gates [ref. 8, Ch.~4].

Arbitrary quantum circuits can be described by a list of gates parametrised by the qubits they operate on. Each quantum computing architecture uses a specific set of gates to implement universal computations. TQEC supports only the definition of CNOTs and the implementation of T, P and V gates, but it does not directly support the application of a Toffoli gate (widely used in classical reversible circuits^6^). In order to implement this gate, it has to be first decomposed into a sequence of TQEC supported gates (Fig. 5). The same applies for the Hadamard (H) gate, which is decomposed as the P, V, P sequence. TQEC supported gates have the following matrix representation where global factors are not included.


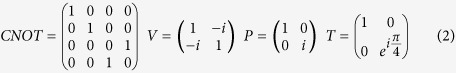


Gate decompositions can be computed dynamically using tools like^7^ or are known beforehand (e.g. the previous Hadamard and Toffoli decompositions). The current synthesis is able to use both methods, but irrespective of the chosen method, the output of this synthesis step is a circuit where all the non-TQEC gates were replaced with their decomposition into TQEC gate sequences.

### ICM Conversion

**Figure 6:**
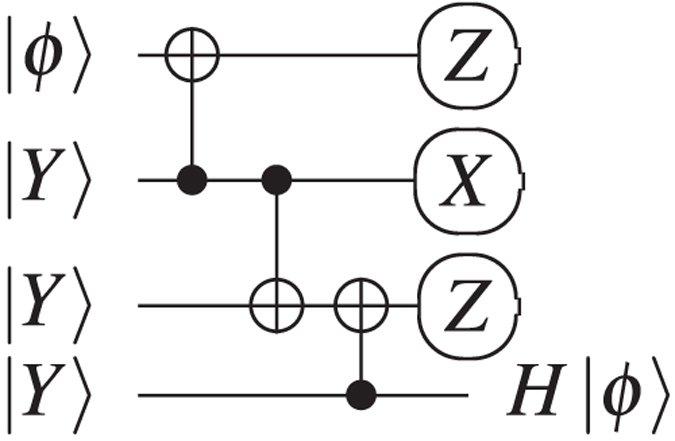
The ICM implementation of the Hadamard gate is the result of executing three gate teleportation circuits (corrections are not illustrated).

TQEC supports the CNOT gate and the rotation gates are implemented using a teleportation circuit^1^ (Fig. 3) with ancillae initialised to 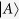 or 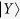. The ancillae states are included through state injection into the TQEC circuit (e.g. Fig. 6).

**Figure 7:**
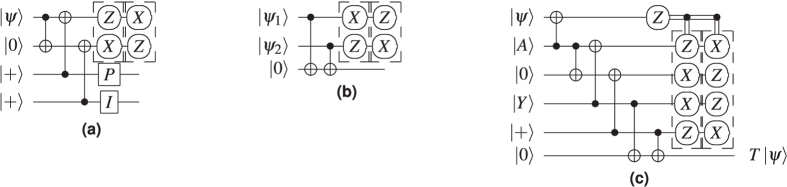
Teleportations: (**a**) Selective destination; (**b**) Selective source^12^ and the combination of the two (**c**), with the *T* gate teleportation circuit (Fig. 3) to produce deterministic circuitry for a *T*-gate. The first column of measurements is chosen if the corrective P gate making use of the injected 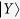 state is required^12^.

Teleported gate applications are probabilistic, meaning that after measurement the output qubit will either need or not need a correction in the form of another quantum gate. The correction requirement is signalled by the measurement result, and some of the corrections are Pauli gates (X, Y, Z), while others are non-Pauli. Pauli gate corrections are not required to be applied directly, because their effect can be tracked through the circuit^9^, and applied only at the end of the computation (at circuit outputs). However, tracking is not possible for non-Pauli corrections, and this is the case for the T gate which requires P gate corrections. The selective source and destination mechanism can be used instead of dynamically modifying the circuit structure to probabilistically accommodate P gates (Fig. 7).

The nature of the teleported gates shows that the structure of an arbitrary TQEC circuit is ICM^10^, because the entire circuit consists of qubit (I)nitialisations, followed only by (C)NOT gates and then by (M)easurements. For this reason, during the second step an ICM circuit is generated starting from the output of the first step (gate decomposition). This procedure is detailed in ref. 10.

### Generating Circuit Geometry

**Figure 8:**
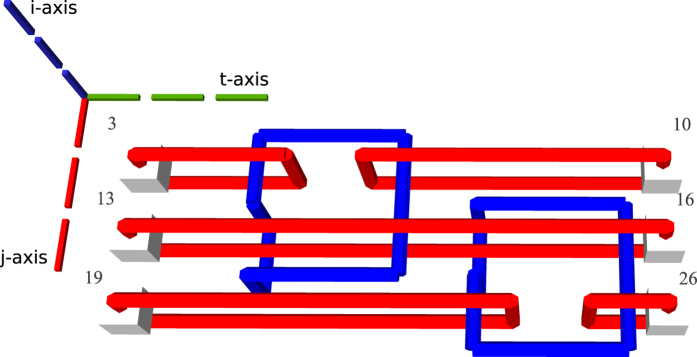
The geometry corresponding to the circuit in Fig. 9b. The light gray cubes numbered 3, 13, 19 denote configurable circuit inputs, and the ones numbered 10, 16, 26 are outputs. Primal defects are red, and dual defects blue. The first horizontal defect pair spanned between the spheres 3 and 10 represent the topmost qubit from Fig. 9b. Each closed dual defect structure represents a CNOT.

The ICM circuit representation synthesised in the previous step is transformed into a three dimensional geometric representation. In order to limit confusion, instead of the x-, y- and z- axis, the i-, j- and t-axis are referenced. A motivation for the choice of the axis names and how a quantum computer will use the geometric representation is offered in the supplementary material. The inputs and the outputs of the circuit are aligned along the j-axis, the gate geometries are depicted along the t-axis. The qubit defects are placed along the i-axis. More exactly, as depicted in Fig. 8, information is processed from the input towards the outputs, and the inputs are at lowest t-axis coordinate while outputs at the highest t-axis coordinate. The first input has the lowest j-axis coordinate, and the last input the highest. Primal input pins of a qubit have the same j-axis and t-axis coordinates, but different i-axis coordinates.

In the subsequent figures representing TQEC geometries, the three dotted lines indicate the axes: green the t-axis, blue the i-axis, and red the j-axis. The common point where the axes are joined indicates the origin of the three dimensional space.

**Figure 9:**
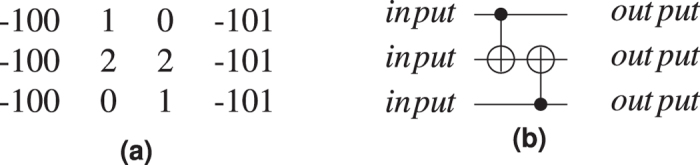
A matrix-like representation of the circuit is used to generate a circuit’s geometry obtained after marking the inputs/outputs accordingly with different values (e.g. −100 and −101), and the controls and the targets with separate values (e.g. 1 and 2). The matrix representation of (**a**) is equivalent to the circuit from (**b**). For each line in the matrix, a defect pair is defined, and a CNOT will have the control/target drawn at the coordinates indicated by the row and the column corresponding to its matrix encoded position. The geometry of a single CNOT circuit is presented in Fig. 10.

When considering the t-axis horizontal and the j-axis vertical, the gates are aligned horizontally and the qubits vertically. A *matrix-like representation* of the circuit, inspired by the quantum circuit formalism, is used: qubits are the lines of the matrix and each CNOT occupies a certain column. Each initialisation and measurement basis is encoded by an integer (e.g. −99 for 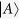 initialisation, and −98 for 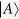 measurement), circuit input outputs marked distinctively (e.g. −100 for input, −101 for output), and the controls and the targets have separate values (e.g. 1 and 2). The geometry is constructed by traversing the matrix column wise, thus drawing the entire sequence of CNOTS starting from circuit inputs towards outputs (Fig. 9).

Due to the direct mapping between the ICM representation, the matrix representation of the circuit and the geometry, the resulting geometry has a single layer format and does not include any *optimisation* like bridging^11^.

### Distillation Scheduler

The injected states required for the construction of teleported rotation gates are the result of distillation procedures^1^ abstracted as boxes. A *scheduler* is a distillation box placement algorithm. A *schedule* can be either *heterogeneous*, when both distillation box types are included, or *homogeneous* when a single box type is present. A schedule expresses the geometric placement of distillation boxes so that these are easily connected to the circuit. For the purpose of the current synthesis method, it was considered that all the distillations have to be ready before the circuit starts being executed. However, each distillation circuit has an associated probability of failure, and additional ones are required in order to have enough ready. In order to ease the connection to the circuit, the obtained schedules have, similarly to the circuit geometry, a single layer format: the boxes are stacked along the i-axis and aligned along the j-axis.

A schedule is computed starting from a list of circuit pin pairs: a distillation box is scheduled for each pair, to ensure the circuit will implement the designed functionality. The type (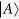 or 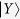) and the coordinates of the injection are known from the geometry generating step. Thus, the type of the scheduled box is identical to the injection’s type, and the geometric position of the box is determined starting from the injection’s coordinates. A homogeneous schedule is the result of a same-type injection pin pairs list and its construction is used in a future step of the synthesis.

Each distillation box includes a pair of output pins, too, and box pins need to be connected to circuit pins. The simplest way to connect boxes to the circuit is if the circuit and the box pins have a common coordinate. Therefore, in the current version of the scheduler, boxes are placed so that their pins have the j-axis coordinate of their circuit counterparts. Each box is three dimensional and if the j-axis distance between two circuit pins is less then the j-axis dimension of a box, then the boxes will be stacked along the i-axis (e.g. Hadamard gate in the Examples section).

Algorithm 1 is sketching the scheduling procedure which takes as inputs a list *L* of injection pin pairs and a list *Dim* of box dimensions. The pseudo code of the algorithms uses partially an object oriented syntax. For example, *l*.*coord*.*j* gets the j-axis coordinate from the coordinates attribute *coord* of object *l*. The algorithm uses an object *R* which represents a two dimensional *region* representing the layer (parallel to the j- and i-axis) where the boxes will be placed in the three dimensional space. For each injection pin pair (*l* ∈ *L*) three parameters are required to schedule a box of type *l*.*type*: the j-axis coordinate of the pins (*j* at Line 5) and the necessary jspan and ispan of the box to be scheduled (*reqj* and *reqi* at Lines 6, 7). The parameters are used to compute the set *A* of possible sub regions of *R* where the box could be placed. The sub region *rmin* with the lowest i-axis coordinate is chosen (Line 9), the sub region is removed from *R* in order to not allow other boxes to be placed there, and finally the box is scheduled at *posj, rmin*.*i*.


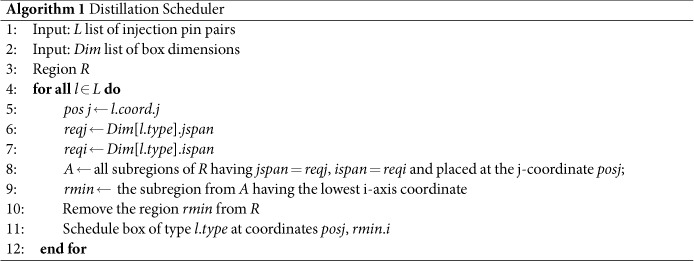


### Distillation Failure Simulator

Spare boxes need to be scheduled, too, and their number is a function of the box type failure probability. Consequently, there will exist a 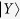 spare boxes schedule and a 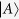 spare boxes schedule. Both are homogeneous schedules obtained after constructing *ghost* injections to the circuit: 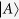 type and 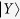 type ghosts. The ghosts are used only to hint to the scheduler where to place the boxes, and are not used or referenced anywhere in the circuit. Once all the distillation boxes were scheduled, the failure of all the boxes (including spares) is simulated using Algorithm 3.

Algorithm 2 generates ghost pins having a large enough distance along the j-axis, so that boxes are not stacked but linearly arranged in a row. The algorithm will generate *n* pin pairs of type *type*, places the first pair at the j-axis coordinate *sw* and subsequent boxes with a j-axis distance of *offj* between. Multiple rows (e.g. *m*) of boxes (similar to stacking) can be obtained by generating ghosts at the same coordinates. After repeating the algorithm *m* times, the output list *L* (Lines 6, 11) of ghost pin pairs will contain *m* times the same *n* pins. The pins of a pair have the same j-axis coordinate, and the t-axis and i-axis coordinates are not computed because they are not relevant (Lines 9, 10) for the scheduler (Algorithm 1). After scheduling the *m* × *n* spare boxes, the homogeneous schedule is organised as an array. This arrangement simplifies the connection between boxes and real (not ghost) circuit pins, because the defect segments will be constructed using straightforward rules.


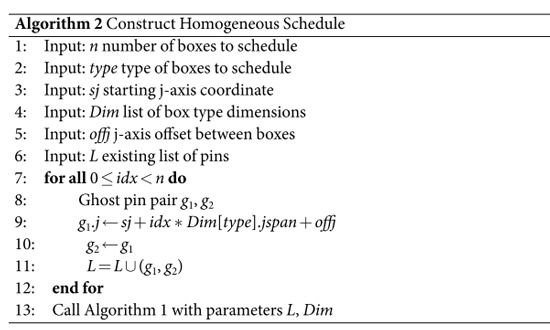


The next problem is to determine which succcessful box should be connected to which circuit injection pin pair (ghosts are not considered). All the boxes of type *t* are stored in a queue *B*_*t*_ and it is assumed that the per box failure probability is *p*. All the type *t* circuit pin pairs are stored in the *Q*_*t*_ queue. The solution is to take the first unused successful box for every pin pair *q* ∈ *Q*_*t*_. The algorithm runs until the queue *B*_*t*_ is empty (Line 5). The failure of each queued box is randomly determined based on *p* (Lines 7–11), and a successful box will be connected to the current circuit pin pair *q* (Line 9).


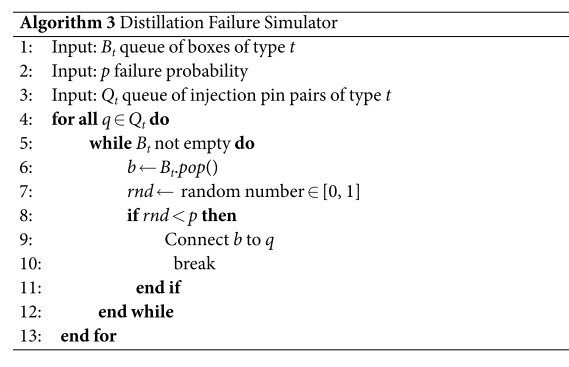


### Connecting Pins

There is a high probability to successfully execute a TQEC circuit after the geometry was generated and enough distillation boxes were scheduled. The final step is to connect distillation boxes to the circuit. Knowing which boxes output high fidelity states, the states are used as injected circuit inputs. In a real-world environment, the successful distillation of states to be injected is known after executing the distillation subcircuits, but the presented synthesis method includes a simulation for determining successful ones.

Scheduling was performed so that connecting the boxes to the circuit would be straightforward. The first consequence was that the schedules have a single layer format. Secondly, in the heterogeneous schedule, boxes have the same j-axis coordinate like the circuit pins, meaning that two orthogonal segments (one along the i-axis and one along the t-axis) are sufficient to build a connection.

Additional homogeneous schedules were obtained by using ghost pins nonexistent in the circuit, and by scheduling boxes in an array. A detail not mentioned before is that scheduling is configurable with respect to: 1) which i-axis coordinate is used for the first scheduled box; 2) how j-axis coordinates are iterated (low-to-high or high-to-low); and 3) how i-axis coordinates are iterated. In conjunction with the array arrangement, the configuration influences the corner, the line and the column directions in which the array is filled with boxes. After placing the spare box schedules around the initial schedule (see, for example, the Toffoli gate in the Examples section), connecting real circuit pins to the spares requires defects consisting of three orthogonal segments.

Algorithm 4 captures the rules of connecting the distillation boxes to the circuit. Pairs of box and circuit pins are resulting after the distillatin failure simulation. The connection algorithm takes as input the set of pin pairs computed by Algorithm 3 and determines the defect segments necessary to connect the pins. Because pins are specified using unit cell coordinates, and by the way the schedules were obtained, it is sufficient to compute the Manhattan distances between box and pin coordinates (Lines 4, 7, 9). For example, the t-axis distance between box pin *b* and circuit pin *c* is *c*.*coord*.*t* − *b*.*coord*.*t*. The distances are used to determine segment end points (e.g. *ep*_1_ and *ep*_2_). The first end point has the coordinates of the box pin (Line 3), and subsequent end points are computed by adding the corresponding distances. A segment is determined by its end point coordinates, and segments of zero length are not constructed.


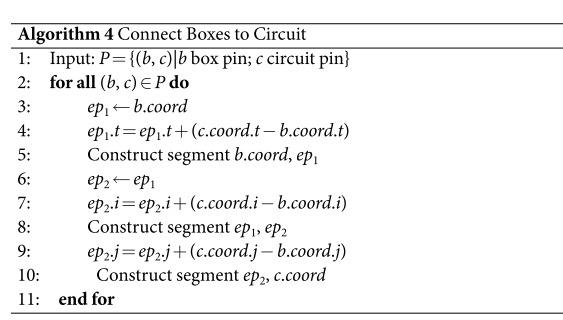


## Examples

The following examples, from a single CNOT circuit to a circuit that implements the Toffoli gate, illustrate the scheduled distillation boxes too. The smaller green boxes represent 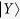 distillations and the larger yellow ones are 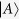 distillations. For the initial schedules (not including spares), the stacking of boxes is visible (e.g. Hadamard gate in Examples section). The array arrangement of the scheduled spares is also noticeable (e.g. Examples section).

### CNOT Gate

**Figure 10:**
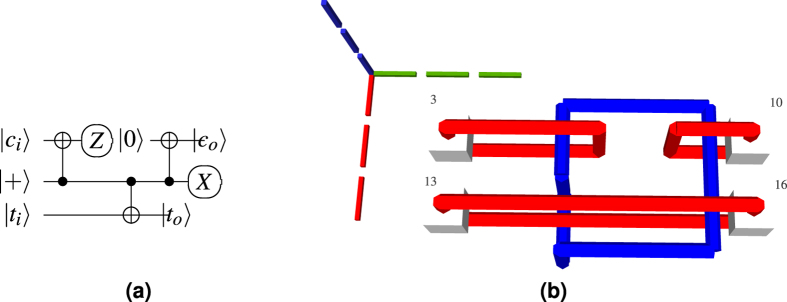
The primal-primal CNOT circuit identity expressed: (**a**) using the quantum circuit formalism; (**b**) as a TQEC circuit geometric description. The qubit initialised into 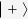 and measured into the *X* basis, in (**a**), represents a TQEC dual qubit (blue) in (**b**), and all the other qubits are primal (red).

A TQEC circuit consisting of a single CNOT has two inputs and two outputs which correspond to the control and the target. Both the control and the target qubits are formed by defect pairs that start/end at the inputs/outputs. It was mentioned that the braid between a dual defect and a primal defect results in the implementation of a logical CNOT. However, it is preferred to have both the control and the target logical qubits of the same type, and this is achieved by using a circuit identity (Fig. 10a). Figure 10 depicts the geometry of a single CNOT circuit implemented through the use of the identity.

### P Gate

**Figure 11:**
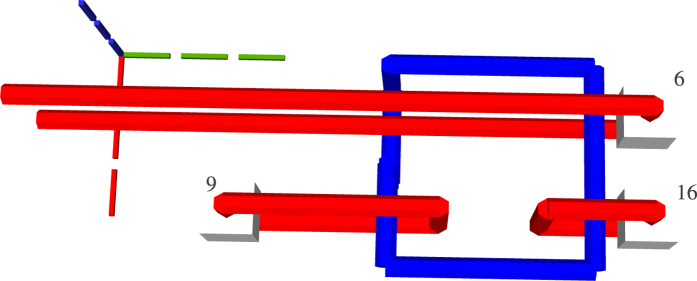
TQEC geometry of the teleported P gate (Fig. 3).

P gates are implemented by the use of teleportation circuits. A corresponding TQEC circuit will consist of an input, an output, an ancilla initialised into the 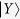 state (the encoding of an injected state) and a CNOT (Fig. 11). The circuit structure is independent of the fidelity of the used 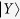 state.

**Figure 12:**
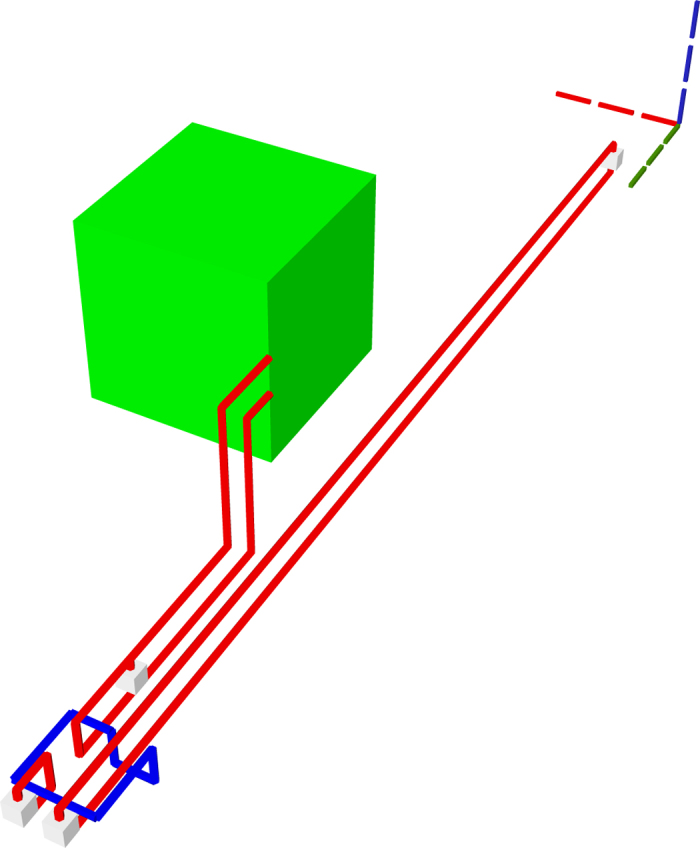
If the boxes have 100% success rate, the schedule of the P gate contains a single |*Y*〉 box.

**Figure 13:**
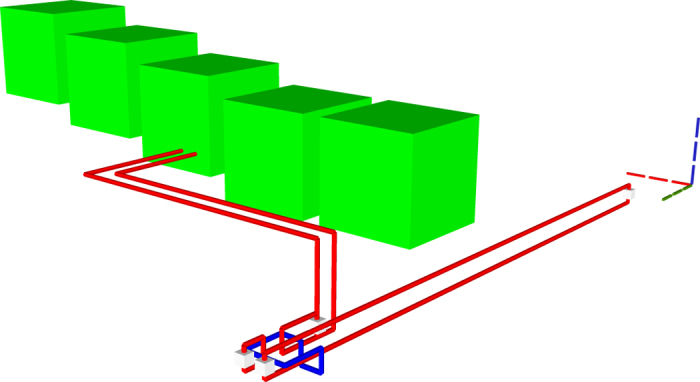
Four |*Y*〉 spare boxes are required when the injected state fidelity needs to be increased and the distillation boxes have a success rate of 80%.

Low fidelity injected states need to be distilled and, assuming that distillations have 100% success rate, a single box would be required (Fig. 12). If the success rate of the box would be lower (e.g. 80%), additional spare distillation boxes need to be scheduled. The resulting homogeneous schedule is placed before the circuit inputs, and after simulating box failures, one of the boxes is connected to the circuit injection ancilla (Fig. 13).

### Hadamard Gate

TQEC circuits are based on topological cluster states, which do not support the direct implementation of a Hadamard gate. One of the solutions is to decompose the gate into a series of implementable gates and to synthesise the circuit description afterwards. Topological cluster states are related to the surface quantum error-correction code^1^, which allows a Hadamard implementation without decomposing the gate. The difference between the capability of the topological cluster states and the surface code stems from the graph state structure of the first: the surface code does not use graph states and is thus more flexible to a certain degree^2^,^14^.

**Figure 14:**
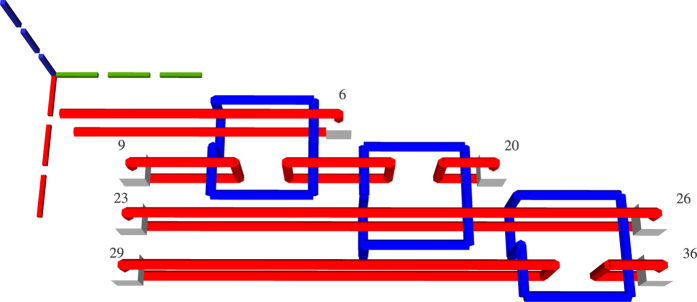
TQEC geometry of the circuit from Fig. 6.

**Figure 15:**
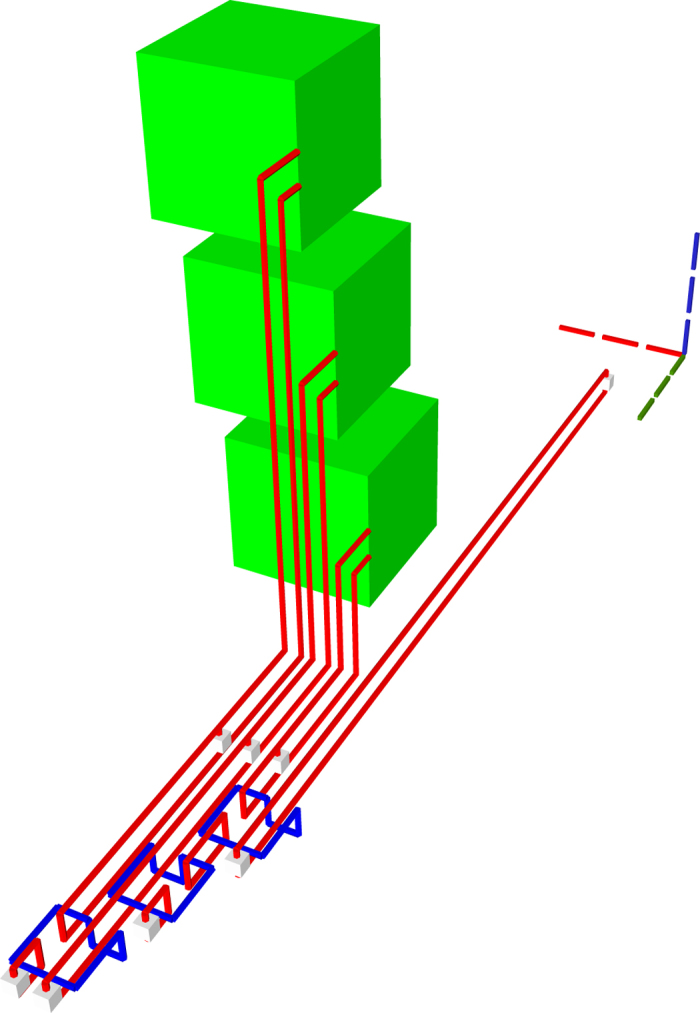
The Hadamard gate schedule contains three |*Y*〉 boxes if all the injected states have high fidelity and the boxes have 100% success rates.

**Figure 16:**
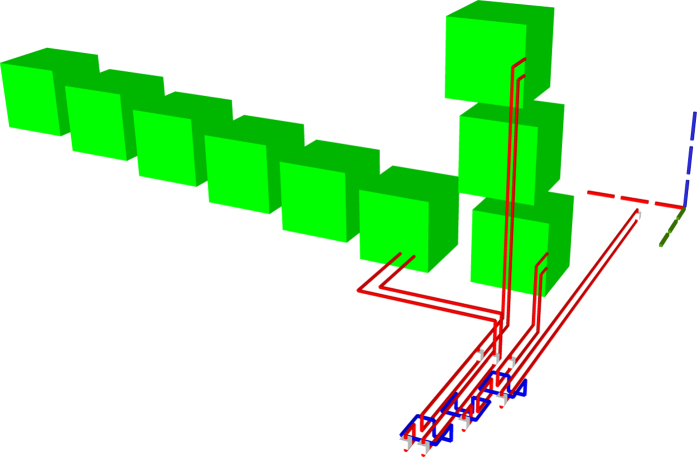
The complete schedule, including spares, is illustrated for the case when the injected state fidelity needs to be increased by using distillation boxes with a success rate of 80%.

A TQEC Hadamard circuit consists of an input, an output, three 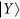 state initialised ancillae and three CNOTs: one for each teleported gate implementation (Fig. 14). Three distillation boxes need to be scheduled in the case of high fidelity states and 100% successful distillations (Fig. 15). The additional spare boxes required to compensate a lower box success rate (e.g. 80%) form a homogeneous schedule positioned on the j-axis before the initial schedule (Fig. 16).

### T Gate

**Figure 17:**
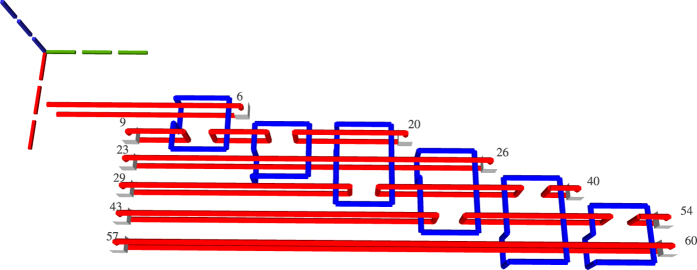
TQEC geometry of the circuit from Fig. 7.

**Figure 18:**
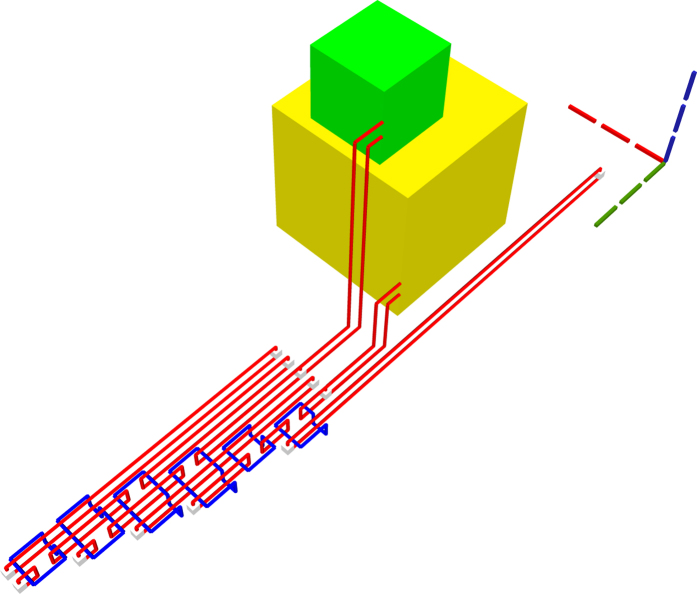
The heterogeneous schedule required for the T gate contains a single |*A*〉 and a single |*Y*〉 box (used for the correcting P gate). This is illustrated for the situation when all the injected states have high fidelity and the boxes have 100% success rates.

**Figure 19:**
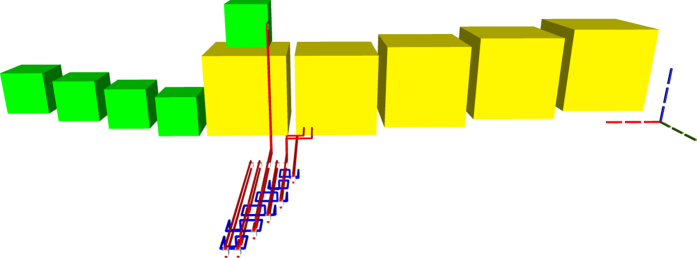
All the scheduled boxes, including spares, are illustrated for the situation when the injected state fidelity needs to be increased by using distillation boxes with a success rate of 80%.

Teleported gate applications are probabilistic. The P and the Hadamard gates allowed tracking the corrections through the circuit, but this not possible for the T gate: P gate corrections need to be applied right after it. A non-probabilistic T gate would have the same circuit structure like the P gate except that instead of 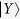 an 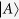 is used. Due to their probabilistic nature and correction requirement, each TQEC T gate has to be followed by a selective source/destination circuit, which can be easily transformed into TQEC geometries (Fig. 17). After considering distillations Figs 18 and 19 are obtained.

### Toffoli Gate

**Figure 20:**
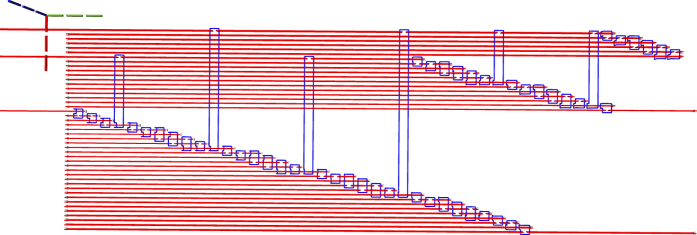
TQEC geometry implementing the Toffoli circuit from Fig. 5. The T and T^†^ gates are implemented using selective source/destination subcircuits (Fig. 7) and the Hadamards are decomposed (Fig. 6).

**Figure 21:**
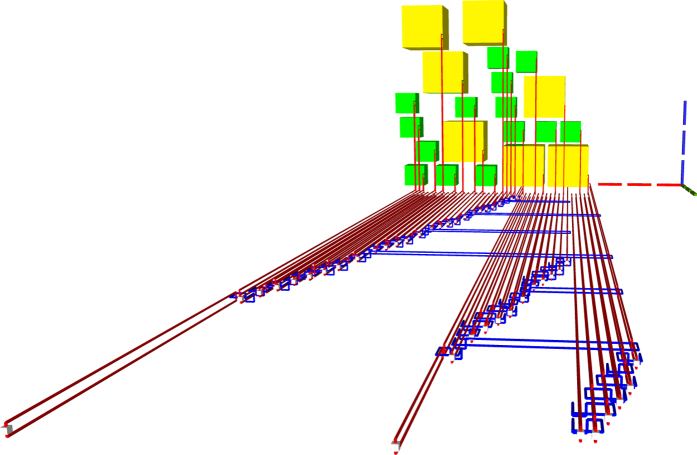
The heterogeneous schedule required for the Toffoli gate implementation is illustrated for the situation when all the injected states have high fidelity and the distillation boxes have 100% success rates. The inputs are at the top of the image, the outputs at the bottom. The scheduled boxes are depicted.

**Figure 22:**
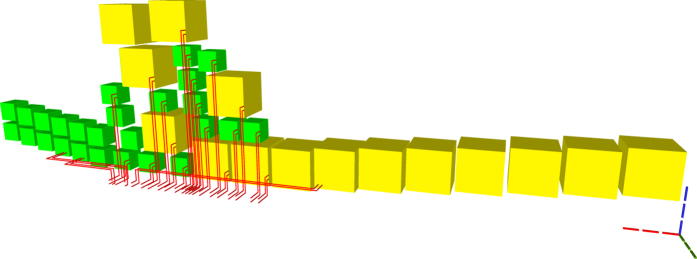
All the scheduled boxes, including spares, are illustrated for the situation when the injected state fidelity needs to be increased by using distillation boxes with a success rate of 80%. The initial schedule (Fig. 21) contained 21 boxes. The homogeneous schedule on the left side contains 12 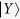 spare boxes, and the schedule on the right contains eight spare 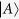 boxes. Through simulation it was determined that four 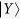 and three 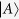 from the initial schedule (middle) fail and, as a result, spares are used.

The TQEC implementation of a Toffoli (Figs 20, 21, 22) gate is the result of synthesising its decomposition (Fig. 5) which consists of seven T gates, two Hadamards and a single P gate. All the gates in this decomposition will be implemented through teleportations.

## Discussion and Future Work

TQEC circuit synthesis is the basis of a complete design stack, which is required to include optimisation and *verification* methods. Circuit optimisation will be used, for example, for reducing the bounding box (geometric volume) of the generated geometry (including schedules), while through verification the optimisation results will be checked for correctness. This section sketches future work directions which are directly related to the herein presented synthesis method.

### Geometric Description

TQEC circuit geometries are assumed to have a single layer format. This is not a requirement, but an implementation decision for the current version, and future synthesis variants could generate geometries extended on multiple layers. Furthermore, it is not required to separate inputs and outputs into two disjoint blocks placed at t-axis opposite coordinates. Inputs and outputs are allowed to have any coordinate, as long as the circuit still implements the designed (correct) functionality.

**Figure 23:**
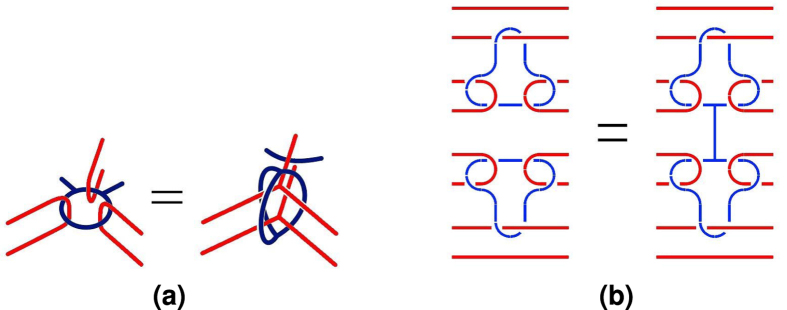
TQEC geometry transformations: (**a**) the cage rule^13^; (**b**) bridging^11^.

**Figure 24:**
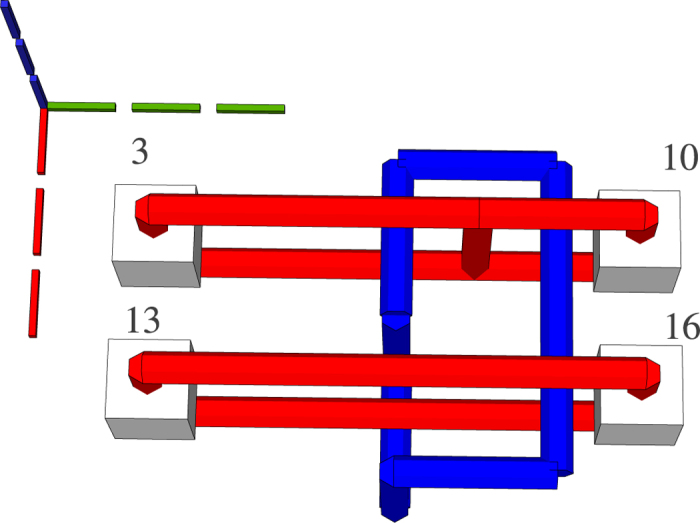
The geometry of a primal-primal CNOT after bridging the primal defect structures representing the control qubit. The initial circuit was illustrated in Fig. 10.

TQEC circuit geometries can be manipulated through circuit identities expressed as geometric transformations (Fig. 23). Instead of generating a non-optimised geometry, this can be synthesised from the beginning using, for example, bridging^11^. That operation does not affect the implemented computation, and has the advantage, for certain types of circuits, of reducing their geometric volume (expressed in volume units, see the introductory section). This is possible by correctly joining certain defects so that no distance exists between them anymore. For example, bridging can be applied to the primal-primal CNOT (Fig. 10) at the primal defects representing the control qubit and the result is a shorter geometry along the t-axis (Fig. 24).

Another topic of interest is to devise automatic geometry compacting algorithms. These will selectively iterate through a set of geometric transformations and determine if the resulting circuit geometry is more optimal with respect to a specified metric. The most general metric is the number of volume units required to represent the geometry (see Glossary in Appendix). Global optimums are very difficult to achieve, but the focus is to firstly research automation, because this was not extensively done up to this point. Geometry optimisation is still performed in the research community by hand, for example^11^, and any degree of automation will result in a major productivity increase.

### Distillation Box Scheduling

The decision to place distillation box schedules at the beginning of the circuit, was taken in order to simplify the synthesis method. Once more, this is not a requirement, and boxes can be placed anywhere in the geometry. A simple optimisation is to move each box along the t-axis right before its output is required. The Toffoli geometry (see Examples section) is a good example: some 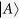 and 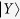 boxes are connected to qubits which are required only at the end of the circuit. A large portion of the defect lengths is not required because the CNOTs are applied late (considering the t-axis as a time axis). Assuming that the CNOT has a starting t-axis coordinate of 200, and a box placed before an input has coordinate 10, it implies that the defects can be shorter and the box can be placed at, for example, 170. If this solution is chosen, the problem of failing distillation boxes and the scheduling of the required set of spares needs to be addressed in a different manner.

The presented scheduling introduced a two dimensional placement of the boxes, and schedules are arranged one next to the other along the j-axis. Scheduling boxes anywhere in the geometry will result in a three dimensional scheduler, and the difficulty would be to determine and implement criteria to still maintain the circuit’s fault-tolerance: given the per box failure probability, are enough boxes available and ready to be connected to the circuit requiring a high fidelity injected state?

### Automatic Defect Construction

Relaxing the assumptions made about geometric construction and box scheduling introduces additional complexities in the automatic construction of connections. The current version utilises a maximum of only three segments to connect an arbitrary box to the corresponding injection pins. A more complex connection mechanism is required if multiple layers are used for the geometry and boxes are allowed to be placed anywhere in the three dimensional space. Electronic design methods use routing algorithms to add wires between the placed circuit components. Lee’s algorithm or channel routing are among the mostly used starting points in classical circuit design, and these could be very well be adapted for the case of TQEC, where defects are the equivalent of classical circuit wires.

## Conclusion

TQEC circuit engineering is a relatively new field of computer engineering and its initial goal is to help construct the first scalable quantum computer. This will be achieved through developing, improving and using circuit design automation tools. Although steps towards constructing a functional TQEC design stack were previously proposed, this work introduced the complete automated synthesis of TQEC circuits. This is the starting point to the study of optimising geometric descriptions, a challenge which can be solved by expanding the TQEC circuit engineering community and by encouraging collaboration within.

The proposed synthesis method consists of a step sequence which includes gate decomposition, fault-tolerant circuit transformation, generation of TQEC circuit geometries, scheduling of specific ancilla state distillation circuits and, finally, connection of disjoint geometric elements into a single TQEC circuit geometric description. Each step was algorithmically formulated, and the synthesis results of the most commonly used quantum gates were presented and analysed. Future work building on the synthesis results, including optimised scheduling of distillations and a more compact geometric description, were also presented and discussed.

## Additional Information

**How to cite this article**: Paler, A. *et al*. Synthesis of Arbitrary Quantum Circuits to Topological Assembly. *Sci. Rep.* **6**, 30600; doi: 10.1038/srep30600 (2016).

## Supplementary Material

Supplementary Information
