# Supplementary material for: Synthesis of Arbitrary Quantum Circuits to Topological Assembly
Source: Sci Rep. 2016 Aug 2;6:30600. doi: 10.1038/srep30600 (PMC4969756; doi:10.1038/srep30600)
Supplement: Supplementary Information [file srep30600-s1.pdf]

Supplemental Material - Synthesis of Arbitrary Quantum Circuits to Topological Assembly

Alexandru Paler[1*], Simon J. Devitt[2], and Austin G. Fowler[3]

[1]Universitatea Transilvania, Facultatea de Matematică si Informatică, Braşov 500091, România
[2]Center for Emergent Matter Sciences, Riken, Saitama 351-0198, Japan
[3]Google Inc., Santa Barbara, California 93117, USA
[*]alexandrupaler@gmail.com

ABSTRACT

Given a quantum algorithm, it is highly nontrivial to devise an efficient sequence of physical gates implementing the algorithm on real hardware and incorporating topological quantum error correction. In this paper, we present a first step towards this goal, focusing on generating correct and simple arrangements of topological structures that correspond to a given quantum circuit and largely neglecting their efficiency. We detail the many challenges that will need to be tackled in the pursuit of efficiency. The software source code can be consulted at https://github.com/alexandrupaler/tqec.

Appendix: Glossary

Box: the bounding box of the *geometric description* of a *distillation circuit*. The type of the box is identical to the state distilled by the distillation circuit. The term is used to also reference distillation sub circuits.

Computational universality: capability of a computing model to capture all of the power of quantum computation.

Configurable input/output: a circuit *input/output* which can be initialised/measured in various bases (e.g. X, Z).

Connection: *defect* spanned between a distillation box *pin* and a circuit pin.

Cube: a subdivision of a *volume unit* representing all the *unit cells* necessary for constructing *defects* with a diameter enabling *topological error-correction* with a specific *distance*.

Defect (geometry): the geometric abstraction of a *lattice* defect. It contains at least one *segment*.

Defect (lattice): a set of physical *face qubits* arranged on neighbouring *unit cells* missing from the *lattice*. The qubits are removed without affecting the connectivity of their neighbourhood by performing Z-basis measurements.

Distance (code): a measure of error-detection and -correction capabilities of a specific code.

Dual (face qubit): *face qubit* of a dual unit cell.

Dual (lattice): the self-similar *lattice* after geometrically translating the one constructed by tiling *unit cells*.

Dual (logical qubit): a *logical qubit* defined in the *dual lattice*.

Dual (unit cell): the unit cell resulting after tiling two primal unit cells along each of the three axis (Fig. 2).

Face qubit: any qubit located at the center of one of the faces of the cubic *unit cell*.

Failure probability (box): the probability that a *distillation circuit* will not output a state with a higher *fidelity*.

Failure probability (gate): the probability that a quantum gate (physical or logical) will not deliver the expected output.

Fault-tolerant threshold: physical gate failure probability below which it is possible to use error correction to perform an arbitrarily large quantum computation arbitrarily reliably.

Fidelity: for our purposes a distance measure between two states u and v both expressed as complex column vectors. A high fidelity is characteristic of states that are close. The fidelity F of a state u is computed by comparing it with an ideal version v using $F = u^{\dagger}v$.

Geometric description: captures all the necessary elements for describing a *TQEC circuit* at the *logical layer*. It consists of the three dimensional coordinates of the *defect segments* and *inputs/outputs*.

Ghost pin: a nonexistent pin, but whose existence is simulated just for allowing the construction of a *schedule*. *Spare boxes* are scheduled using ghost pins, but are connected when necessary to *real pins*.

Graph state: a specific quantum state derived from a graph of vertices and edges by associating a qubit prepared in $|+\rangle$ with each vertex and applying a CZ gate to every pair of qubits associated with the end points of every edge.

Heterogeneous (schedule): a *schedule* computed for *boxes* of different types.

Homogeneous (schedule): a *schedule* computed for *boxes* of the same type.

ICM circuit: a quantum circuit consisting only of qubit initialisation, CNOT gates and qubit measurements. The circuits are useful for representing fault-tolerant *universal computations*.

Input/output (circuit): the interface of a circuit that is used to either input a state to be processed or to output a resulting state. Accordingly, each *logical qubit* has an input (used for initialisation) and an output (used for measurement). For *TQEC circuits* the inputs/outputs abstract *physical qubit* coordinates from the *lattice*.

Lattice: a highly regular structure resulting after tiling *unit cells*) along the three axes of a three dimensional space. Its resulting structure is of a *graph state* where *physical qubits* are entangled according to the unit cell pattern. Each qubit can be associated with an integer three dimensional coordinate. The lattice includes two self-similar sub lattices: itself (*primal*) and a version of itself which is geometrically translated by the vector $(1,1,1)$ (*dual*). By convention, the unit cells forming an initial lattice are considered primal.

Logical layer: an abstraction of the *physical layer* where multiple entities from the physical layer are used for defining a single logical entity with better characteristics. For example, *logical qubits* with lower failure probabilities are constructed out of multiple physical qubits each with higher failure probabilities. Another example is the logical CNOT gate which is the result of braiding *defects*, an abstraction involving many physical operations.

Logical qubit: two *defects* each abstracted in the *geometric description* by a sequences of *segment* end points.

Matrix-like circuit representation: the result of mapping the circuit representation to an array of integers. Each integer represents a different element of the circuit (e.g. -100 for inputs, -101 for outputs, 1 for CNOT control and 2 for CNOT target).

Optimisation (TQEC circuit): the process of reducing the bounding box of a *geometric description* corresponding to a *TQEC circuit*.

Physical layer: one of the layers at which error corrected computational models operate. The physical layer is an abstraction of the hardware, where entities existing at this layer mostly have a direct hardware equivalent. For example, *physical qubits* are direct abstractions of the hardware representing and/or operating a qubit (e.g. photon).

Physical qubit: the quantum state represented and manipulated by a single physical quantum hardware entity.

Pin: the three dimensional coordinate of a *box* or circuit interface. Two pins exist for each box or circuit *input/output* because their coordinates indicate where a connection has to be attached. A *connection* is a geometric abstraction of a *defect*, and because information is manipulated as *logical qubits* there are two *connections* required for each box or circuit input/output.

Pin pair: the two *pins* associated to the same input/output.

Primal (face qubit): *face qubit* of a *primal unit cell*.

Primal (lattice): *lattice*.

Primal (logical qubit): a *logical qubit* defined in the *primal lattice*.

Primal (unit cell): a *unit cell* used for constructing the lattice necessary for *TQEC circuits*.

Real pin: *pin*.

Region: a rectangular two dimensional space extracted from a three dimensional space by constraining two coordinates to intervals and the third coordinate to a particular value. Regions are used for placing the *geometric description* and the *schedules*.

Rotation gate: arbitrary single qubit gate. The most utilised rotation gates are T, P and V because they are sufficient for achieving *computational universality*. In TQEC these are implemented through teleportation circuits which use injected states.

Schedule: list of three dimensional space coordinates where *boxes* are to be placed.

Scheduler: algorithm that places *boxes* in a three dimensional space.

Segment (connection): part of a *connection*. It is geometrically abstracted by a line segment.

Segment (defect): sub set of *physical qubits*, from the *defect*, which would exist on co-linear *unit cells* if the *lattice* is drawn in a three dimensional space.

Spare box: bounding box of a potential additionally required *distillation circuit*. Spares are used because distillation circuits have an associated *failure probability* and a failed box cannot be used for the implementation of a TQEC *rotation gate*.

State distillation: circuit that takes multiple low *fidelity* instances of the same state and outputs a single higher fidelity state.

State injection: process of encoding a specific rotated state of a lattice *physical qubit* ($|A\rangle$ or $|Y\rangle$) at the *logical layer*. The resulting *logical qubit* has two *defects* originating from the physical qubit. The latter does not belong to any of the defects. For this reason, both defects have a pyramid structure with its tip at the three dimensional coordinate of the physical qubit.

Synthesis (circuit): process of transforming an algorithmic description into a circuit description. For the case of TQEC, the *logical layer* circuit description is the *geometric description*.

Topological assembly: the set of elements representing a *TQEC circuit* specified by a *geometric description*.

Topological cluster state: the quantum state represented as *graph state* having the structure of a *lattice*.

Topological error-correction: a class of quantum error-correction procedures based on *topological cluster states*.

TQEC circuit: Topological Quantum Error Corrected circuit. For this work, an *ICM circuit*.

Unit cell: a particular *graph state* shown in 2. A single cell contains 18 *physical qubits* such that six are entangled with four neighbours and 12 qubits are entangled with two neighbours. In an infinite three dimensional tiling of unit cells, every qubit is entangled with four neighbours and each unit cell contributes six qubits.

Verification (TQEC circuit): process of checking if a specified *geometric description* has the structure required for implementing a particular quantum computation.

Volume unit: a cubic structure consisting of 125 *cubes* used for measuring the volume of a *geometric description* in a manner which is error-correction code independent.

Appendix: Geometrical Description Volume

Bounding boxes were initially introduced when referring to the distillation subcircuits, and mentioned in the context of optimising a synthesised geometric description. However, the metric of bounding boxes was not introduced and discussed accordingly. This section presents the *equivalent volume*[7] of a geometric description and the considerations backing its definition.

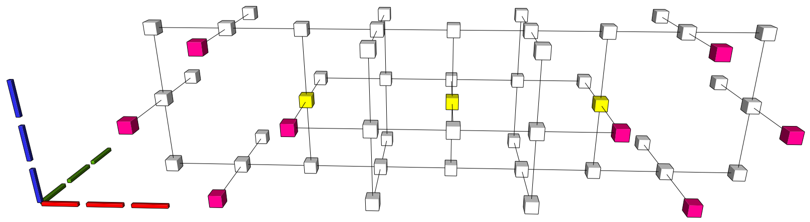

Figure 25. A lattice of $3 \times 1 \times 1$ primal unit cells where two single cell defects were defined. The ring of qubits marked pink (length = 4) and the chain of qubits marked yellow (length = 3) are used for error-correction.

TQEC circuits use a quantum error-correction procedure based on topological cluster states in which information is encoded by constructing defects and operated on by braiding the defects. Error-correction is based on logical operators which are defined as rings of specific physical qubits around defects and specific physical qubit chains connecting defects. The overall code distance is the minimum value between the shortest ring and the shortest chain. The shortest ring is found at the defect with the smallest diameter, and the shortest chain exists between the closest defects. Fig. 25 depicts the way rings and chains are determined when considering the lattice layout: each unit cell on the defect margin contributes a physical qubits to the ring diameter d_f, each unit cell along the distance between two defects contributes a physical qubit to the chain length d_d. The perimeter p_f of a ring is $4d_f$, where d_f equals the number of unit cells along the diameter, because the defects are considered to have quadratic cross-sections. In order to have a code distance of p_f, the shortest chain has to have a length greater than p_f: $d_d = 4d_f + 1$ is achieved when two defects are at least $4d_f$ unit cells apart. Therefore, in general, it can be argued, that in order to use an error-correction with distance $4d$, the defects are required to have a diameter of d unit cells and need to be at least $4d$ unit cells apart. Because the code distance is a function of physical failure rates, diameters and defect distances need to be increased for high failure rates.

The general error-detection procedure is detailed in[1,11] and for the purpose of this section it suffices to mention that it uses the parities of lattice unit cells: there are six face qubits on each unit cell, and their X basis measurement parity is even in the absence of errors. An error is detected on odd parity cells, and correction is performed after choosing pairs of odd parity cells as end points of even parity unit cell chains.

Apart from the previous discussion, the minimal distance (three) is achieved when the diameter is one unit cell wide, and each defect is two unit cells apart from another. Therefore, the definition of a logical qubit requires at least four unit cells from the i,j-plane of a three dimensional lattice. Considering the configurable geometries at input/outputs, the minimal length of a defect connecting an input to an output will be four unit cells long. However, such a construction is not fault-tolerant[11], because errors on the qubits measured in the Z basis cannot be detected and could propagate in an uncontrolled manner (*cascade*). Fault-tolerance is achieved after increasing the defect diameters and using the error-correction method presented in[11]: consider the chain of pairs of neighbouring five-sided dual unit cells sharing Z-measured qubits nearest to the defect border. Thus, the minimal defect diameter required to implement fault-tolerant TQEC is of two unit cells (2×2 unit cells, $d_f = 2$). The code distance will be $d = 8$, requiring at least eight unit cells between any two defects ($d_d = 9$). Thus, the $1/4$ ratio between diameter and distance is again obtained.

It is simpler to use a volume measure which is code distance independent, instead of counting the number of lattice unit cells required to execute a geometrically described TQEC computation. For this reason, a cubic *volume unit*, a standardised lattice with fixed dimensions, is introduced (Fig. 26). The volume unit is obtained by tiling cubes instead of unit cells, where a

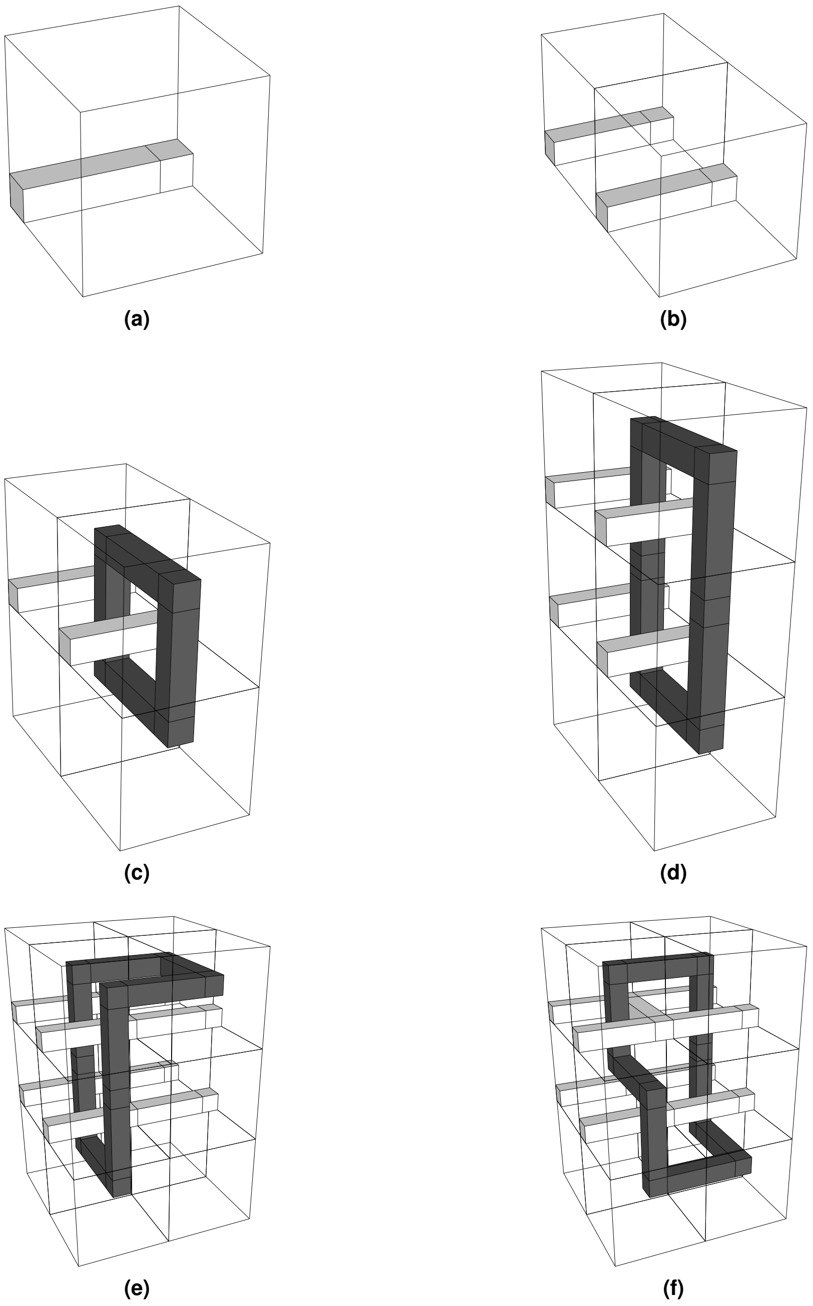

Figure 26. A volume unit consists of 5^3 primal cubes (very light gray): a) a five boxes long defect occupies a volume unit; b) two primal defects require two volume units; c) a dual circular defect (dark gray) around a single primal defect; d) a dual circular defect around two primal defects; d) the necessary volume is doubled after deforming the dual defect and extending the primal defects; e) twelve volume units are required for the bridged CNOT.

cube with sides of length d contains d^3 primal unit cells. Therefore, the complete lattice required for executing a TQEC circuit is the result of tiling volume units, which contain an exact number of cubes, which in turn are three dimensional tilings of unit cells. Volume units and cubes are intermediate abstractions between a complete lattice and unit cells.

A volume unit contains $(1+4)^3 = 5^3$ cubes having the necessary diameter for defect construction so that distance $4d$ error-correction is possible. Cubes are formed of primal unit cells but, the definition could be extended, after observing that a cube of dual unit cells is obtained in the middle of a $2 \times 2 \times 2$ cube arrangement. Tiling cubes has the same effect as tiling unit cells (Fig. 2).

In a volume unit there are enough cubes so that a primal defect having three non-parallel segments could be defined at the margins. Two volume units are the minimum for defining two defects parallel in the same i,j plane. It is not possible to construct a circular dual defect around any of the defects without increasing the lattice to $1 \times 2 \times 2$ volume units. A dual defect around both primal defects requires a lattice of $1 \times 2 \times 3$ volume units. Implementing the bridge CNOT (cf. Fig. 24 and 26) requires a lattice of 12 volume units due to the $2 \times 2 \times 3$ necessary arrangement.

As a conclusion, the geometric description captures the computation (ICM circuit) being implemented into TQEC circuits, and is independent of the code distance. However, in the previous sections it was always mentioned that segment end point coordinates are specified using unit cell coordinates. For geometries implemented using a distance four error-correction this is true, as each defect is only a single unit cell wide. For codes with larger distances, those definitions do not hold for unit cells. If one replaces every occurrence of unit cell with the term cube, the geometric description is valid for arbitrary error-correction procedures using topological cluster states. A single aspect remains to be discussed: the effect of code distance scaling on defect construction. For the distance four code, a cube includes a single unit cell. Scaling the code distance by two, a cube will contain eight unit cells, and each cube face will consist of four unit cell faces. Therefore, when a segment end point is specified having the coordinate of cube and code distance is scaled by two, four unit cell face qubits from the same cube face are measured in the Z basis.

Appendix: Using a Geometrical Description

The geometric description of a synthesised TQEC circuit is a high-level description of the operations a fault-tolerant quantum computer needs to execute. This section explains why the i- and j-axis coordinates can be understood as hardware entity addresses, and the t-axis coordinate as the time when the entities are to be used. The execution of a geometrically described computation starts by mapping the description to an empty lattice[12], determining the bases of physical qubit measurement bases and then operating the quantum hardware. There are multiple justifications why the latter operation is performed by gradually constructing and measuring the lattice.

The first explanation is related to the complexity of TQEC circuits. Circuit depth is a measure of complexity for classical and quantum circuits, and it represents the longest path, expressed usually in terms of number of gates, between any input and output of the circuit. The depth of a geometry is not considered an appropriate measure of the underlying ICM circuit complexity. This is because, ICM circuits are Clifford+T[6] instances, and the relevant complexity in executing such circuits is generated by the number of T gates. It could be argued that the depth of an ICM circuit can be defined as the maximum number of T gates between an input and an output. The T gates on any circuit path introduce a temporal ordering: the sequence in which gates have to be executed. In terms of ICM circuit, this would correspond to an ordered sequence of (M)easurements because gates are implemented through teleportations; gate execution is finalised only after corresponding qubits were measured. Moreover, in ICM circuits, the T gate ordering is equivalent to the ordering of the probabilistic P gate corrections necessary for teleported T gates. If any of these corrections is left out, the executed computation is not correct. The correction mechanism was briefly mentioned and illustrated using the circuits from Fig. 3.

TQEC geometric descriptions were synthesised so that inputs have the lowest and outputs have the highest t-axis coordinate, while braids and measurements have intermediary coordinates. For example, in Fig. 20, the measurements corresponding to selective source and destination subcircuits follow on the t-axis immediately after the braids. It can be also observed that the ordering of the measurements has a geometric representation: for the gates on the same wire in Fig. 5, the corresponding measurements have increasing t-and j-axis coordinates. The j-coordinate increases because of the additional ancillae introduced during ICM transformation, while the t-coordinate is just a reflection of the gate ordering.

The second justification for which the lattice is gradually constructed and measured is related to a quantum hardware optimisation problem. If the complete lattice would be constructed and measured afterwards, a very large number of quantum hardware entities corresponding to physical qubits would be required. Anyway, as previously explained, there is an order in which the measurements have to be executed, meaning that on average each hardware entity would be required to maintain the states of the physical qubits for a long time. This requirement has a threefold problematic: firstly, it is very difficult to build hardware able to correctly maintain states for long times; secondly, even if the hardware would support this, the cost of building such a high number of entities would be prohibitive; and thirdly why should the physical qubits be initiated that soon if their states are unused for a long time?

The solution to the above problem uses a layered approach: the geometry is sliced along the t-axis into a sequence of primal and dual layers (Fig. 28), and the number of required hardware entities is trimmed to just the one necessary to manipulate states from two subsequent layers. The highly regular structure of the lattice offers the support for obtaining the slices. Each logical qubit measurement is specified in terms of physical qubit coordinates: the i- and j- axes coordinates are quantum hardware entity addresses and the t-axis coordinate indicates the time when the hardware is initialised, entangled with neighbouring entities and measured.

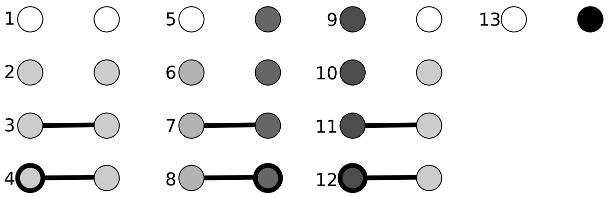

Figure 27. Implementing an arbitrary rotation gate decomposed into three single qubit rotated measurements using two qubits. Colour coding: white - uninitialised state; black - output state; very light gray - initialised qubit; intermediate levels of gray - intermediate output states obtained after each rotated measurement of one of the qubits; measured qubit - thick black outline. At time step 1 both qubits are not initialised. At time steps 2 and 3, the qubits are initialised and entangled. The first measurement is performed on the leftmost qubit at step 4. The sequence is repeated again twice, until at time 13 the rightmost qubit has the output state of the rotation gate.

Constructing and measuring the layers according to their t-axis coordinate will effectively enforce the ordering of the gates from the ICM circuit. The suitability of the layered approach is based on the observation that the execution of a TQEC circuit encoded into a topological cluster state is the execution of a measurement-based quantum computation[13], where the measurement basis of individual physical qubits is a function of the computation implemented at the higher level (logical layer). The method is similar to how a linear graph state (e.g. Fig. 27) would be measured to implement a rotation gate: decompose the rotation in a time ordered measurement sequence (X,Z,X basis rotated measurements), and execute the measurement sequence.

Considering that a lattice is sliced then the number of primal layers is larger by one compared to the number of dual layers. Assuming that the layer sequence is $\{p_0, d_0, p_1, d_1, \ldots, d_{n-1}, p_n\}$, a quantum computer executing a TQEC geometric description will execute the following steps: 1) initialise and entangle p_0 and d_0, for $i = 0$; 2) measure the primal layer p_i; 3) initialise the primal layer p_{i+1} and entangle it to the dual layer d_i; 4) measure the dual layer d_i; 5) reinitialise the dual layer d_{i+1} and entangle it to the primal layer p_{i+1}; 6) increase i by one; 7) go to step 2 until no primal layer is left ($i = n$).

The structure of the layers is illustrated in Fig. 28. Primal layers have the physical qubits arranged according to Fig. 28d, and dual layers similar to Fig. 28e. In the process of entangling layers the physical qubits with the same i-, j-coordinates and sequential t-coordinates are entangled (e.g. the layer from Fig. 28d will be entangled to the one from Fig. 28e). As a consequence, the gradual construction and measurement of layers is respecting the structure imposed by tiling unit cells along three dimensions.

References

1. Fowler, A. G., Mariantoni, M., Martinis, J. M. & Cleland, A. N. Surface codes: Towards practical large-scale quantum computation. *Physical Review A* **86**, 032324 (2012).
2. Paler, A., Devitt, S., Nemoto, K. & Polian, I. Software-based pauli tracking in fault-tolerant quantum circuits. In *Design, Automation and Test in Europe Conference and Exhibition (DATE), 2014*, 1–4 (IEEE, 2014).
3. Paler, A., Devitt, S., Nemoto, K. & Polian, I. Synthesis of topological quantum circuits. In *Proceedings of the 2012 IEEE/ACM International Symposium on Nanoscale Architectures*, 181–187 (ACM, 2012).
4. Wille, R., Große, D., Miller, D. M. & Drechsler, R. Equivalence checking of reversible circuits. In *Multiple-Valued Logic, 2009. ISMVL'09. 39th International Symposium on*, 324–330 (IEEE, 2009).
5. Nielsen, M. A. & Chuang, I. L. *Quantum computation and quantum information* (Cambridge university press, 2010).
6. Paler, A., Polian, I., Nemoto, K. & Devitt, S. J. A fully fault-tolerant representation of quantum circuits. In *Reversible Computation*, 139–154 (Springer, 2015).
7. Fowler, A. G. & Devitt, S. J. A bridge to lower overhead quantum computation. *arxiv* **1209** (2012).
8. Raussendorf, R., Harrington, J. & Goyal, K. Topological fault-tolerance in cluster state quantum computation. *New Journal of Physics* **9**, 199 (2007).
9. Ross, N. J. & Selinger, P. Optimal ancilla-free clifford+ t approximation of z-rotations. *arXiv preprint arXiv:1403.2975* (2014).
10. Fowler, A. G. Time-optimal quantum computation. *arXiv preprint arXiv:1210.4626* (2012).
11. Fowler, A. G. & Goyal, K. Topological cluster state quantum computing. *Quantum Information & Computation* **9**, 721–738 (2009).

12. Paler, A., Devitt, S. J., Nemoto, K. & Polian, I. Mapping of topological quantum circuits to physical hardware. *Scientific reports* **4** (2014).

13. Childs, A. M., Leung, D. W. & Nielsen, M. A. Unified derivations of measurement-based schemes for quantum computation. *Physical Review A* **71**, 032318 (2005).

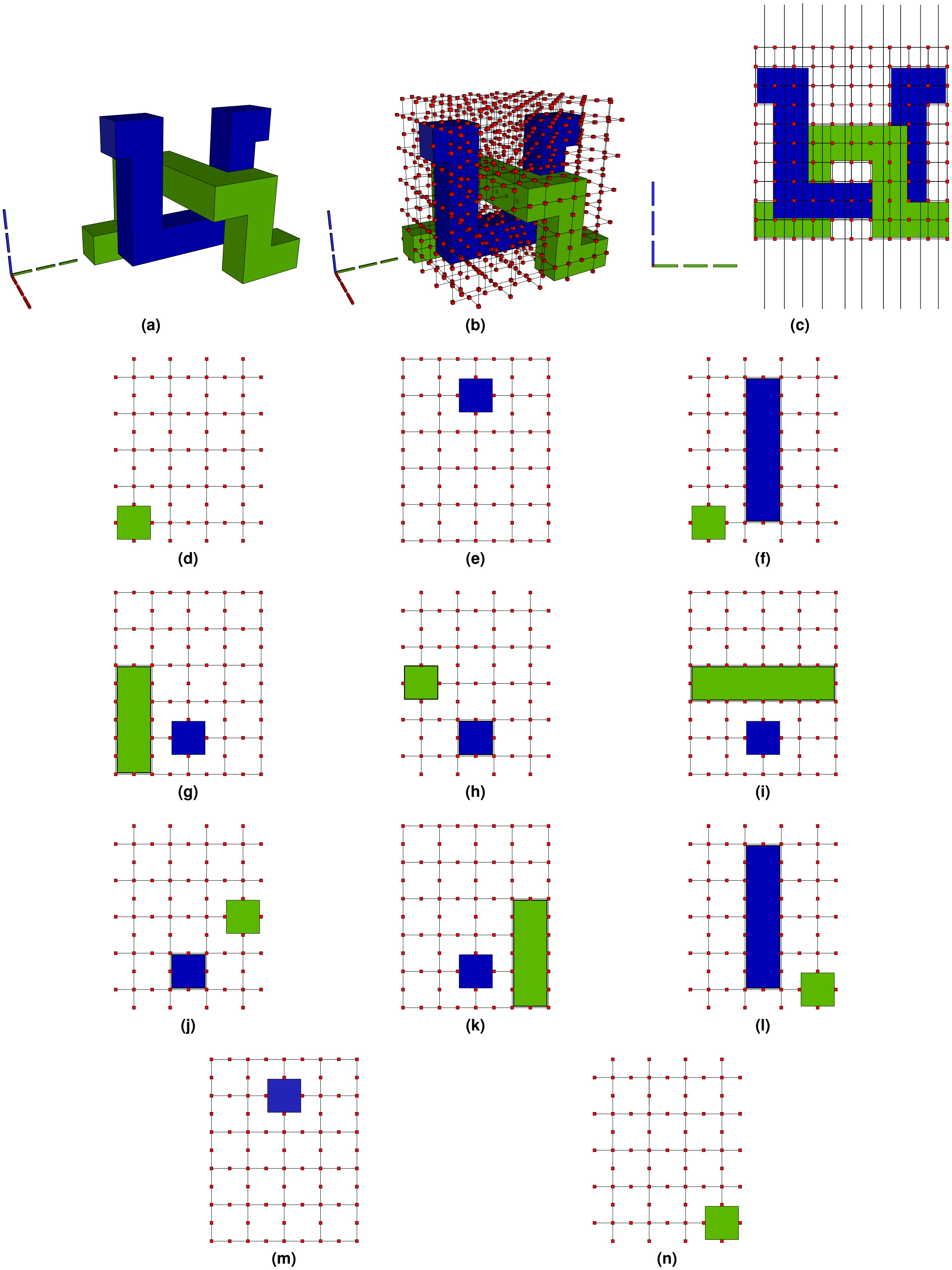

Figure 28. A primal defect (green) braided with a dual defect (blue): a) defect structure; b) defects superimposed on a lattice; c) j-axis view of the lattice having the slice positions indicated with parallel vertical lines; d-n) sequence of lattice slices.
